# Regional Differences in Mortality Rates and Characteristics of Decedents With Hepatitis B Listed as a Cause of Death, United States, 2000-2019

**DOI:** 10.1001/jamanetworkopen.2022.19170

**Published:** 2022-06-28

**Authors:** Kathleen N. Ly, Shaoman Yin, Philip R. Spradling

**Affiliations:** 1 Division of Viral Hepatitis, National Center for HIV, Viral Hepatitis, STD, and TB Prevention, Centers for Disease Control and Prevention, Atlanta, Georgia

## Abstract

**Importance:**

US hepatitis B mortality has been described nationally, but examination subnationally may identify differences in mortality rates and decedent characteristics, including birthplace.

**Objective:**

To examine characteristics of decedents with hepatitis B–listed deaths during 2010 to 2019 and compare age-adjusted hepatitis B–listed death rates during 2010 to 2019 vs 2000 to 2009.

**Design, Setting, and Participants:**

This cross-sectional study used Multiple Cause of Death data from 50 US states and the District of Columbia (DC) from 2000 to 2019 to assess characteristics of US residents with hepatitis B listed as an underlying cause of death (UCOD) or contributing cause of death on death certificates. Data were analyzed from September 2019 to May 2022.

**Exposures:**

Hepatitis B listed as underlying or contributing cause of death.

**Main Outcomes and Measures:**

Outcomes of interest were hepatitis B–listed death counts, age-adjusted rates, and characteristics of decedents during 2000 to 2019. The distribution of hepatitis B–listed deaths according to sociodemographic characteristics and UCOD among US- and non-US–born decedents were also examined.

**Results:**

A total of 35 280 decedents with hepatitis B listed as the cause of death were identified, including 17 483 deaths during 2010 to 2019. Decedents were 63.3% US-born, and 25.8% of decedents were Asian or Pacific Islander and 46.5% of decedents were White; 28.4% of decedents were listed as having hepatitis C virus (HCV) or HIV coinfection. State-level rates significantly surpassed the overall US rate (0.47 deaths per 100 000 population) in DC (high, 1.78 deaths per 100 000 population), Hawaii, Oklahoma, California, Tennessee, West Virginia, Mississippi, Oregon, Washington, Louisiana, Kentucky, and New York (low, 0.61 deaths per 100 000 population). Median (IQR) age at hepatitis B death was significantly younger in Kentucky (54.0 [46.0-64.0] years), West Virginia (56.0 [47.0-65.0] years), Tennessee (57.0 [50.0-65.0] years), Mississippi (58.0 [50.0-65.0] years), and Ohio (59.0 [50.0-66.0] years) than the national median (60.0 [53.0-69.0] years), which itself was significantly younger than nonhepatitis B–listed deaths (77 [63.0-87.0] years; *P* < .001). Hepatitis B was the UCOD among approximately 30% of US- and non-US–born decedents with hepatitis B COD. Irrespective of birthplace, most decedents had liver-related UCOD. Compared with non-US–born decedents, US-born decedents more frequently had nonliver conditions listed as UCOD. Liver cancer was the predominant UCOD among non-US–born decedents (37.9% of decedents). From 2000 to 2009 compared with 2010 to 2019, the hepatitis B–listed mortality rate significantly decreased nationally (change, −18.97%) and in 14 states; significant increases were observed in West Virginia (change, 83.78%) and Kentucky (change, 69.44%).

**Conclusions and Relevance:**

These findings suggest that US-born decedents constituted two-thirds of all hepatitis B–listed deaths and median age at death was youngest in Appalachian states. Irrespective of birthplace, most decedents had liver-related UCOD; however, US-born decedents more frequently had nonliver UCOD than non-US–born decedents. In addition to addressing liver-related complications, US-born persons with chronic infection may also require diagnosis and management of multiple comorbidities.

## Key Points

**Question:**

Were there regional differences in mortality rates and characteristics among decedents with hepatitis B listed as a cause of death in the US during 2000 to 2019?

**Findings:**

In this nationwide cross-sectional study including 35 280 decedents from 2000 to 2019, the highest hepatitis B–listed death rates were observed in coastal and Appalachian states; in addition, younger median age at death occurred predominantly in Appalachian states. Most decedents, regardless of birthplace, had liver-related conditions listed as underlying cause of death, and decedents born in the US, who constituted approximately two-thirds of all deaths, more frequently had nonliver conditions listed as underlying cause of death compared with non-US–born decedents.

**Meaning:**

These findings suggest that in addition to addressing liver-related complications, US-born persons with chronic infection may also require diagnosis and management of multiple comorbidities.

## Teaser

This cross-sectional study assesses characteristics of US decedents with hepatitis B–listed deaths during 2010 to 2019 and compares rates with 2000 to 2009.

## Introduction

Chronic hepatitis B can lead to cirrhosis and hepatocellular carcinoma, resulting in premature death. In addition to increased mortality from liver-related causes, chronic hepatitis B has been associated with premature mortality and elevated mortality rates from all causes.^1,2,3,4^ Elevated mortality and premature death among persons with chronic hepatitis B has been associated with coinfection with hepatitis C virus (HCV), HIV, or hepatitis D virus (HDV), diabetes, metabolic syndrome, alcohol use disorder, and smoking.^5,6,7,8,9,10,11^

National hepatitis B death rates were relatively constant during 1999 to 2019 and highest among decedents aged 55 years and older, non-Hispanic Asian and Pacific Islander persons, and men.^12,13^ Annual national viral hepatitis surveillance reports have included hepatitis B mortality estimates since 2004, displaying state-level data since 2015.^13^ However, these reports lacked information on decedent place of birth, comorbidities, and underlying vs contributing causes of death. In 2019, states with the highest hepatitis B–listed death rates included Hawaii, Oklahoma, Tennessee, and Oregon. US Multiple Cause of Death (MCOD) data are compiled from all US-registered deaths that are housed in each state’s vital registration office. Demographic information, such as state of residence, US- vs non-US birthplace, age, race and ethnicity, and sex are available in MCOD data and are highly complete.

In 2021, the Department of Health and Human Services (DHHS) published a national strategy for eliminating hepatitis B as a public health threat in the United States.^14^ The plan described core indicators to measure progress, including a 65% reduction in the rate of hepatitis B deaths by 2030. One specific goal of the plan was to “improve public health surveillance through data collection, case reporting, and investigation at the national, state, tribal, local, and territorial health department levels.”^14^ State and local health departments were encouraged to develop chronic hepatitis B surveillance programs, including understanding hepatitis B mortality in their jurisdiction, and to prioritize activities according to the needs of the populations they serve.

Accordingly, we examined MCOD data for decedents with hepatitis B listed as an underlying or contributing COD during 2000 to 2019 to understand subnational variability in death counts and rates, characteristics of decedents (eg, US- vs non-US birthplace and coinfection status), and changes in death rates. Recent US hepatitis B prevalence estimates reported higher numbers and rates of chronic hepatitis B among non-US–born persons compared with those born in the US.^15,16^ In the most recent analysis of the National Health and Nutrition Examination Survey, persons of Asian descent constituted 69.7% of chronic hepatitis B cases.^15^ Compared with US-born persons, non-US–born persons typically acquire infection earlier in life, which can affect clinical outcomes, including a 25% lifetime risk of premature mortality from liver failure or liver cancer.^17,18,19^ We surmised that, compared with US-born decedents, more non-US–born decedents would have hepatitis B listed as the underlying COD (UCOD). To further explore differences between US- and non-US–born decedents with hepatitis B–listed deaths, we examined the distribution of these deaths by sociodemographic characteristics, median age at death, and UCOD.

## Methods

This cross-sectional study was deemed exempt from institutional review board review and informed consent because all data were obtained from secondary sources without personally identifiable information, per HHS Code of Federal Regulations Title 45 Part 46 2018. This study was reported according to the Strengthening the Reporting of Observational Studies in Epidemiology (STROBE) reporting guideline.

### Data Source

This study used restricted-use US MCOD data that were acquired through approval of a project determination to the Centers for Disease Control and Prevention National Center for Health Statistics (NCHS) research review committee to obtain information on decedent state of residence and US birthplace status.^20^ Through the National Vital Statistics program, NCHS compiles death certificate information from state vital statistics offices to produce national MCOD data.

### Definitions

Region- and state-level hepatitis B–listed deaths included decedents whose residence was in the 50 US states and the District of Columbia (DC). Regions were grouped according to the assignments under the 10 DHHS regional offices.^21^

We defined hepatitis B–listed deaths through *International Statistical Classification of Diseases and Related Health Problems, Tenth Revision* (*ICD-10*)^22^ COD codes if at least 1 code indicative of hepatitis B (ie, B16, B17.0, B18.0, and B18.1) was listed as the UCOD or a contributing COD (CCOD) in the record axis fields. We captured the UCODs and all CCODs to ensure completeness.^2,23,24^ If a decedent had hepatitis B listed as a COD, we looked for evidence of coinfection with HCV (B17.1 and B18.2), HIV (B20-B24), and HDV (B16.0, B16.1, B17.0, and B18.0) present as any COD. A US-born decedent was defined as a birth in the 50 states or DC. A non-US–born decedent was defined as a birth in any country outside of the United States.

### Statistical Analysis

Hepatitis B–listed death counts were enumerated by aggregating data into two 10-year time intervals (2000-2009 and 2010-2019) to improve reliability of state-level measures. Hepatitis B–listed death rates in 2000 to 2009 and 2010 to 2019 were calculated by dividing the number of hepatitis B–listed deaths in a state of residence and time period by the equivalent Census population. Death rates were adjusted to the age distribution of the 2000 US standard population using the direct method. To estimate the variance of age-adjusted death rates, 95% CIs were calculated based on a γ distribution, as developed by Fay and Feuer.^25^ To examine variability in rates during 2010 to 2019, we derived death rate ratios (DRRs) to compare hepatitis B–listed death rates in DHHS regions and states to that of the United States as a whole, denoted by DRR = (*Rate_Region_* or *Rate_State_*)/*Rate_US_*). *Z*-score *P* values were examined to determine if differences in death rates were statistically significant for each region and state compared with the United States and 2010 to 2019 compared with 2000 to 2009. We were unable to calculate hepatitis B–listed death rates by US birthplace status owing to lack of such information in the US Census population data sets. Race and ethnicity information is reported by the funeral director by questioning the decedent’s next of kin or direct observation (including Hispanic, non-Hispanic American Indian or Alaska Native, non-Hispanic Asian or Pacific Islander, non-Hispanic Black, and non-Hispanic White).^26^ Race and ethnicity data were included to describe race and ethnicity disparities by overall, US birthplace status, and coinfection status.

Median age at hepatitis B–listed death, distribution of the status of US birthplace, and HCV, HIV, or HDV coinfection during 2010 to 2019 were examined by state. The Kruskal-Wallis test was used to assess statistically significant differences in median age at hepatitis B–listed death for each region and state relative to the median age at hepatitis B–listed death for the United States. The Pearson χ^2^ test and 95% CIs of proportions were used to determine significant differences in the proportion of hepatitis B–listed deaths among US-born and non-US–born individuals and who had HCV, HIV, or HDV coinfection listed and did not have coinfection listed for each state relative to that proportion of hepatitis B–listed deaths for the United States.

We determined whether there were differences according to US vs non-US birth status by examining the distribution and median age of hepatitis B–listed deaths by sociodemographic and UCOD categories during 2010 to 2019. Among decedents for whom hepatitis B was not listed as the UCOD (ie, hepatitis B instead was listed as a CCOD), we classified the UCOD into 15 broader categories (eTable in the Supplement). Finally, we compared the median age at death of decedents without hepatitis B listed as a COD to the median age at death of US-born and non-US–born decedents with hepatitis B COD by UCOD categories.

We followed NCHS’s policy regarding decedent confidentiality; therefore, we did not display or interpret any subnational results where counts were fewer than 10 deaths. All mortality rates are expressed per 100 000 population. To control for type I errors arising from multiple comparisons, we adjusted *P* values using the step-down Bonferroni method of Holm for any comparisons of state or region with the United States during 2010 to 2019 and rates for any region or state from 2010 to 2019 vs 2000 to 2009.^27^ All *P* values were 2-sided, and we considered *P* < .05 statistically significant. Statistical analyses were performed using SAS software, version 9.4 (SAS Institute) and the map was generated using R Studio, version 1.3.1056 (R Project for Statistical Computing). Data were analyzed from September 2019 to May 2022.

## Results

### Hepatitis B–Listed Deaths During 2010-2019

From 2000 to 2019, there were 35 280 decedents with hepatitis B listed as the cause of death, including 17 797 deaths from 2000 to 2009 (age-adjusted rate, 0.58 [95% CI, 0.58-0.59] deaths per 100 000 population) and 17 483 deaths from 2010 to 2019 (age-adjusted death rate, 0.47 [95% CI, 0.46-0.48] deaths per 100 000 population) (Table 1). Hepatitis B–listed deaths were mostly among men (12 779 decedents [73.1%]), individuals aged 45 to 64 years (9605 decedents [54.9%]), individuals born during 1945 to 1965 (10 849 decedents [62.1%]), and US-born individuals (10 823 decedents [63.3%]). Approximately one-fourth of decedents (4488 decedents [25.8%]) were non-Hispanic Asian or Pacific Islander, 3312 decedents (19.1%) were among non-Hispanic Black individuals, and 8083 decedents (46.5%) were among non-Hispanic White individuals. Among hepatitis B–listed deaths, 4297 decedents (24.6%) had HCV coinfection, 961 decedents (5.5%) had HIV coinfection, and 295 decedents (1.7%) had both HCV and HIV coinfection; 6 deaths (<0.1%) included HDV infection (eFigure 1 in the Supplement).

**Table 1.** Hepatitis B–Listed Deaths by State of Residence in the United States, 2000 to 2009 and 2010 to 2019^a^

Jurisdiction	2000-2009 Deaths		2010-2019 Deaths						2010-2019 vs 2000-2009	
	No.	Rate (95% CI)^b^	No.	Rate (95% CI)^b^	DRR (95% CI)^c^	*P* value^d^^,^^e^	Age at death, median (IQR), y	*P* value^e^^,^^f^	Rate change, %	*P* value^e^^,^^g^
United States	17 797	0.58 (0.58-0.59)	17 483	0.47 (0.46-0.48)	1 [Reference]	NA	60.0 (53.0-69.0)	NA	−18.97	<.001
Region 1										
Overall	758	0.49 (0.45-0.52)	651	0.36 (0.33-0.39)	0.76 (0.70-0.82)	.001	61.0 (54.0-70.0)	.82	−26.53	.001
Connecticut	187	0.48 (0.42-0.56)	116	0.26 (0.21-0.31)	0.54 (0.45-0.66)	.005	61.5 (55.0-71.0)	>.99	−45.83	.005
Maine	46	0.30 (0.22-0.40)	52	0.30 (0.22-0.40)	0.63 (0.47-0.83)	.02	58.0 (50.5-65.5)	>.99	0.00	>.99
Massachusetts	352	0.52 (0.46-0.57)	337	0.42 (0.37-0.47)	0.88 (0.79-0.99)	.28	62.0 (54.0-70.0)	>.99	−19.23	.19
New Hampshire	42	0.29 (0.21-0.40)	35	0.22 (0.15-0.31)	0.46 (0.33-0.66)	.005	59.0 (48.0-72.0)	>.99	−24.14	>.99
Rhode Island	101	0.86 (0.70-1.05)	83	0.63 (0.50-0.79)	1.33 (1.06-1.66)	.14	61.0 (54.0-67.0)	>.99	−26.74	.74
Vermont	30	0.44 (0.29-0.64)	28	0.34 (0.22-0.52)	0.72 (0.49-1.06)	.58	60.5 (53.0-65.5)	>.99	−22.73	>.99
Region 2										
Overall	1986	0.67 (0.64-0.70)	1843	0.55 (0.52-0.58)	1.16 (1.11-1.22)	.001	61.0 (53.0-69.0)	.82	−17.91	.001
New Jersey	468	0.50 (0.46-0.55)	438	0.41 (0.37-0.45)	0.87 (0.79-0.96)	.06	61.0 (54.0-69.0)	>.99	−18.00	.07
New York	1518	0.75 (0.71-0.79)	1405	0.61 (0.58-0.65)	1.30 (1.23-1.37)	.005	61.0 (53.0-69.0)	>.99	−18.67	.005
Region 3										
Overall	1550	0.50 (0.47-0.52)	1351	0.37 (0.35-0.39)	0.78 (0.74-0.83)	.001	59.0 (51.0-66.0)	.001	−26.00	.001
Delaware	46	0.52 (0.38-0.69)	49	0.43 (0.32-0.58)	0.91 (0.68-1.22)	>.99	58.0 (51.0-64.0)	>.99	−17.31	>.99
District of Columbia	74	1.29 (1.01-1.63)	118	1.78 (1.47-2.14)	3.76 (3.13-4.52)	.005	59.0 (54.0-68.0)	>.99	37.98	.74
Maryland	433	0.75 (0.68-0.83)	336	0.49 (0.43-0.54)	1.03 (0.92-1.15)	>.99	58.5 (51.0-68.0)	.43	−34.67	.005
Pennsylvania	605	0.44 (0.40-0.47)	419	0.26 (0.24-0.29)	0.56 (0.50-0.62)	.005	60.0 (52.0-66.0)	>.99	−40.91	.005
Virginia	317	0.40 (0.36-0.45)	282	0.29 (0.26-0.33)	0.62 (0.55-0.70)	.005	59.0 (52.0-66.0)	>.99	−27.50	.005
West Virginia	75	0.37 (0.29-0.46)	147	0.68 (0.57-0.80)	1.44 (1.21-1.70)	.005	56.0 (47.0-65.0)	.005	83.78	.005
Region 4										
Overall	3192	0.53 (0.51-0.55)	3405	0.45 (0.44-0.47)	0.96 (0.92-0.99)	.02	59.0 (51.0-67.0)	.001	−15.09	.001
Alabama	213	0.45 (0.39-0.51)	178	0.32 (0.28-0.38)	0.69 (0.59-0.80)	.005	58.0 (50.0-65.0)	.13	−28.89	.06
Florida	1068	0.54 (0.50-0.57)	1089	0.42 (0.40-0.45)	0.90 (0.84-0.96)	.02	60.0 (53.0-70.0)	>.99	−22.22	.005
Georgia	419	0.48 (0.44-0.53)	411	0.37 (0.34-0.41)	0.79 (0.71-0.87)	.005	59.0 (52.0-67.0)	>.99	−22.92	.007
Kentucky	157	0.36 (0.30-0.42)	299	0.61 (0.54-0.69)	1.30 (1.16-1.46)	.005	54.0 (46.0-64.0)	.005	69.44	.005
Mississippi	152	0.52 (0.44-0.62)	213	0.65 (0.56-0.75)	1.37 (1.20-1.58)	.005	58.0 (50.0-65.0)	.02	25.00	.99
North Carolina	488	0.54 (0.49-0.59)	404	0.34 (0.30-0.38)	0.72 (0.66-0.80)	.005	60.0 (52.5-68.0)	>.99	−37.04	.005
South Carolina	252	0.56 (0.49-0.63)	264	0.45 (0.39-0.51)	0.94 (0.83-1.07)	>.99	59.0 (53.0-65.5)	>.99	−19.64	.31
Tennessee	443	0.71 (0.64-0.78)	547	0.72 (0.66-0.79)	1.53 (1.40-1.67)	.005	57.0 (50.0-65.0)	.005	1.41	>.99
Region 5										
Overall	1851	0.35 (0.33-0.36)	1861	0.30 (0.29-0.32)	0.64 (0.61-0.67)	.001	60.0 (53.0-68.0)	.32	−14.29	.001
Illinois	364	0.28 (0.26-0.32)	315	0.21 (0.19-0.24)	0.45 (0.40-0.51)	.005	60.0 (53.0-71.0)	>.99	−25.00	.007
Indiana	198	0.31 (0.26-0.35)	232	0.30 (0.26-0.35)	0.64 (0.56-0.74)	.005	58.0 (53.0-67.0)	>.99	−3.23	>.99
Michigan	404	0.38 (0.34-0.42)	353	0.28 (0.25-0.31)	0.60 (0.54-0.66)	.005	61.0 (55.0-68.0)	>.99	−26.32	.005
Minnesota	204	0.39 (0.33-0.44)	258	0.40 (0.36-0.46)	0.86 (0.76-0.97)	.19	60.0 (53.0-67.0)	>.99	2.56	>.99
Ohio	526	0.42 (0.39-0.46)	549	0.40 (0.37-0.44)	0.85 (0.78-0.93)	.005	59.0 (50.0-66.0)	.005	−4.76	>.99
Wisconsin	155	0.26 (0.22-0.31)	154	0.22 (0.19-0.26)	0.47 (0.40-0.56)	.005	61.0 (53.0-70.0)	>.99	−15.38	>.99
Region 6										
Overall	2415	0.70 (0.67-0.73)	2348	0.54 (0.52-0.56)	1.14 (1.09-1.19)	.001	59.0 (52.0-67.0)	.001	−22.86	.001
Arkansas	186	0.64 (0.55-0.74)	160	0.47 (0.40-0.55)	1.00 (0.85-1.17)	>.99	57.0 (51.0-66.0)	.38	−26.56	.14
Louisiana	377	0.83 (0.75-0.92)	333	0.61 (0.55-0.68)	1.30 (1.16-1.45)	.005	58.0 (53.0-64.0)	.13	−26.51	.005
New Mexico	95	0.49 (0.40-0.60)	71	0.30 (0.23-0.38)	0.63 (0.49-0.80)	.005	60.0 (53.0-70.0)	>.99	−38.78	.05
Oklahoma	314	0.86 (0.76-0.96)	384	0.88 (0.80-0.98)	1.88 (1.69-2.08)	.005	59.0 (52.0-66.0)	.29	2.33	>.99
Texas	1443	0.68 (0.65-0.72)	1400	0.50 (0.48-0.53)	1.07 (1.01-1.13)	.19	60.0 (53.0-68.0)	>.99	−26.47	.005
Region 7										
Overall	495	0.35 (0.32-0.39)	503	0.31 (0.28-0.34)	0.66 (0.60-0.72)	.001	60.0 (52.0-67.0)	.29	−11.43	.04
Iowa	94	0.30 (0.24-0.37)	130	0.35 (0.29-0.42)	0.75 (0.63-0.90)	.02	60.0 (52.0-67.0)	>.99	16.67	>.99
Kansas	110	0.39 (0.32-0.47)	96	0.29 (0.23-0.35)	0.61 (0.49-0.74)	.005	60.0 (53.0-65.0)	>.99	−25.64	.61
Missouri	217	0.35 (0.30-0.40)	206	0.29 (0.25-0.34)	0.62 (0.54-0.71)	.005	60.0 (51.0-67.0)	>.99	−17.14	>.99
Nebraska	74	0.40 (0.32-0.51)	71	0.34 (0.26-0.43)	0.71 (0.56-0.90)	.07	58.0 (50.0-67.0)	>.99	−15.00	>.99
Region 8										
Overall	373	0.38 (0.34-0.42)	387	0.31 (0.28-0.34)	0.65 (0.59-0.72)	.001	61.0 (54.0-68.0)	.82	−18.42	.01
Colorado	224	0.48 (0.42-0.55)	247	0.41 (0.36-0.47)	0.88 (0.77-0.99)	.33	62.0 (54.0-71.0)	>.99	−14.58	>.99
Montana	30	0.29 (0.20-0.42)	20	0.14 (0.08-0.23)	0.30 (0.19-0.47)	.005	63.5 (55.0-70.5)	>.99	−51.72	.35
North Dakota	10	0.14 (0.07-0.27)^h^	10	0.14 (0.07-0.27)^h^	NA	NA	55.0 (37.0-66.0)	>.99	NA	NA
South Dakota	20	0.24 (0.14-0.37)	13	0.11 (0.06-0.20)^h^	NA	NA	59.0 (58.0-61.0)	>.99	NA	NA
Utah	70	0.35 (0.28-0.45)	78	0.29 (0.23-0.36)	0.62 (0.49-0.77)	.005	58.0 (52.0-65.0)	>.99	−17.14	>.99
Wyoming	19	0.36 (0.22-0.58)^h^	19	0.26 (0.16-0.43)^h^	NA	NA	63.0 (56.0-66.0)	>.99	NA	NA
Region 9										
Overall	4266	0.98 (0.95-1.00)	4227	0.76 (0.74-0.79)	1.62 (1.57-1.68)	.001	63.0 (55.0-72.0)	.001	−22.45	.001
Arizona	255	0.44 (0.39-0.50)	288	0.36 (0.32-0.41)	0.77 (0.68-0.87)	.005	61.0 (53.0-68.0)	>.99	−18.18	.54
California	3713	1.09 (1.06-1.13)	3573	0.85 (0.82-0.88)	1.80 (1.73-1.86)	.005	63.0 (55.0-73.0)	.005	−22.02	.005
Hawaii	189	1.35 (1.16-1.56)	201	1.19 (1.03-1.38)	2.53 (2.19-2.92)	.005	61.0 (54.0-71.0)	>.99	−11.85	>.99
Nevada	109	0.44 (0.36-0.54)	165	0.50 (0.43-0.59)	1.06 (0.91-1.24)	>.99	60.0 (53.0-69.0)	>.99	13.64	>.99
Region 10										
Overall	911	0.73 (0.68-0.78)	907	0.57 (0.53-0.61)	1.21 (1.13-1.30)	.001	61.0 (54.0-68.0)	.82	−21.92	.001
Alaska	51	0.92 (0.67-1.27)	47	0.63 (0.45-0.86)	1.33 (0.98-1.79)	.44	59.0 (51.0-66.0)	>.99	−31.52	>.99
Idaho	40	0.29 (0.21-0.40)	41	0.21 (0.15-0.29)	0.45 (0.33-0.62)	.005	60.0 (56.0-66.0)	>.99	−27.59	>.99
Oregon	302	0.77 (0.68-0.86)	310	0.64 (0.57-0.72)	1.35 (1.20-1.52)	.005	60.0 (52.0-67.0)	>.99	−16.88	.58
Washington	518	0.79 (0.73-0.87)	509	0.61 (0.56-0.66)	1.29 (1.18-1.41)	.005	62.0 (55.0-69.0)	>.99	−22.78	.005

Abbreviations: DHHS, US Department of Health and Human Services; DRR, death rate ratio; NA, not applicable.

^a^ Data Source: 2000-2019 US Multiple Cause of Death data, National Vital Statistics System.

^b^ Death rates are per 100 000 population and adjusted to the age distribution of the 2000 US standard population.

^c^ DRRs were calculated to compare age-adjusted hepatitis B–listed death rates in DHHS regions and states with the age-adjusted hepatitis B–listed death rate among all US residents, denoted by DRR = (*Rate_Region_* or *Rate_State_*) / *Rate_US_*.

^d^ Compared with the US age-adjusted hepatitis B–listed death rate using *Z*-score.

^e^ To control for type I errors arising from multiple comparisons, *P* values were adjusted using the step-down Bonferroni method of Holm for any comparisons of state or region with the United States during 2010 to 2019 and rates for any state or region from 2010 to 2019 vs 2000 to 2009.

^f^ Compared with the national median age at hepatitis B–listed death in US residents using Kruskal-Wallace test.

^g^ Compared age-adjusted hepatitis B–listed death rates during 2010 to 2019 vs 2000 to 2009 using *Z*-score.

^h^ Rates where death counts were less than 20 are considered statistically unstable.

The number of hepatitis B–listed deaths in 13 states exceeded the national mean number of all hepatitis B–listed deaths (mean [SD] 343 [550] deaths per state) (Table 1). California alone accounted for 20.4% of all hepatitis B–listed deaths, followed by New York (8.0%), Texas (8.0%), and Florida (6.2%), with more than 1000 deaths each, followed by (in descending order of death count) Ohio, Tennessee, Washington, New Jersey, Pennsylvania, Georgia, North Carolina, Oklahoma, and Michigan. The cumulative death counts in these 13 states accounted for 65.7% of all hepatitis B–listed deaths.

**Figure 1.**
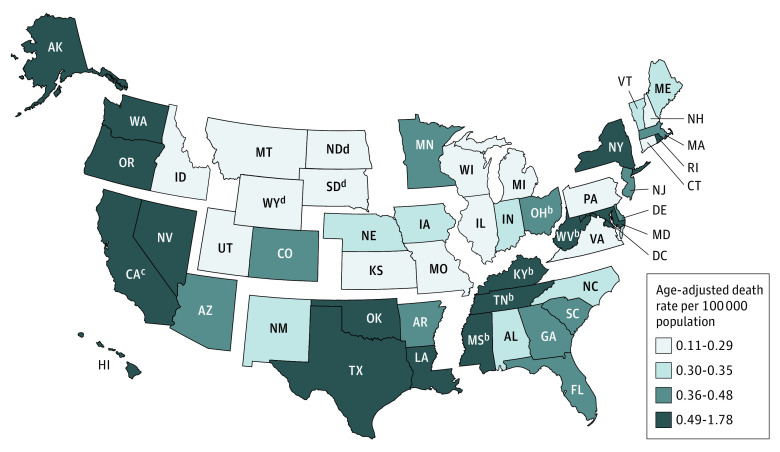
Age-Adjusted Hepatitis B–Listed Death Rates in 50 States and the District of Columbia, Segmented by Department of Health and Human Services Regions, United States, 2010-2019^a^ Data are shown by decedent residence in the US states and the District of Columbia. Data Source: 2010-2019 US Multiple Cause of Death data, National Vital Statistics System. ^a^The hepatitis B–listed death rate for the United States in 2010 to 2019 was 0.47 (95% CI, 0.46–0.48) deaths per 100 000 population. ^b^Significantly younger median age at hepatitis B–listed death than the median age at hepatitis B–listed death for the United States (60.0 years). ^c^Significantly older median age at hepatitis B–listed death than the median age at hepatitis B–listed death for the United States. ^d^Rates where death counts were fewer than 20 are considered statistically unstable.

States in which the hepatitis B–listed death rates significantly surpassed the national hepatitis B–listed death rate included DC (high, 1.78 [95% CI, 1.47-2.14] deaths per 100 000 population), Hawaii, Oklahoma, California, Tennessee, West Virginia, Mississippi, Oregon, Washington, Louisiana, Kentucky, and New York (low, 0.61 [95% CI, 0.58-0.65] deaths per 100 000 population) (Table 1 and Figure 1). The highest death rates were concentrated in the coastal and Appalachian regions (Figure 1). The hepatitis B–listed death rate was the lowest (with respect to statistical reliability) in Montana (0.14 [95% CI, 0.08-0.23] deaths per 100 000 population) followed by Idaho (0.21 [95% CI, 0.15 to 0.29] deaths per 100 000 population), Illinois (0.21 [95% CI, 0.19 to 0.24] deaths per 100 000 population), New Hampshire (0.22 [95% CI, 0.15-0.31] deaths per 100 000 population), and Wisconsin (0.22 [95% CI, 0.19-0.26] deaths per 100 000 population).

The median (IQR) age at hepatitis B–listed death was 60.0 (53.0-69.0) years for the United States (Table 1). Significantly younger median (IQR) ages than the national median age at hepatitis B–listed death were in Kentucky (54.0 [46.0-64.0] years), West Virginia (56.0 [47.0-65.0] years), Tennessee (57.0 [50.0-65.0] years), Mississippi (58.0 [50.0-65.0] years), and Ohio (59.0 [50.0-66.0] years) (Table 1 and Figure 1). A significantly older median (IQR) age at hepatitis B–listed death was observed in California (63.0 [55.0-73.0] years).

**Figure 2.**
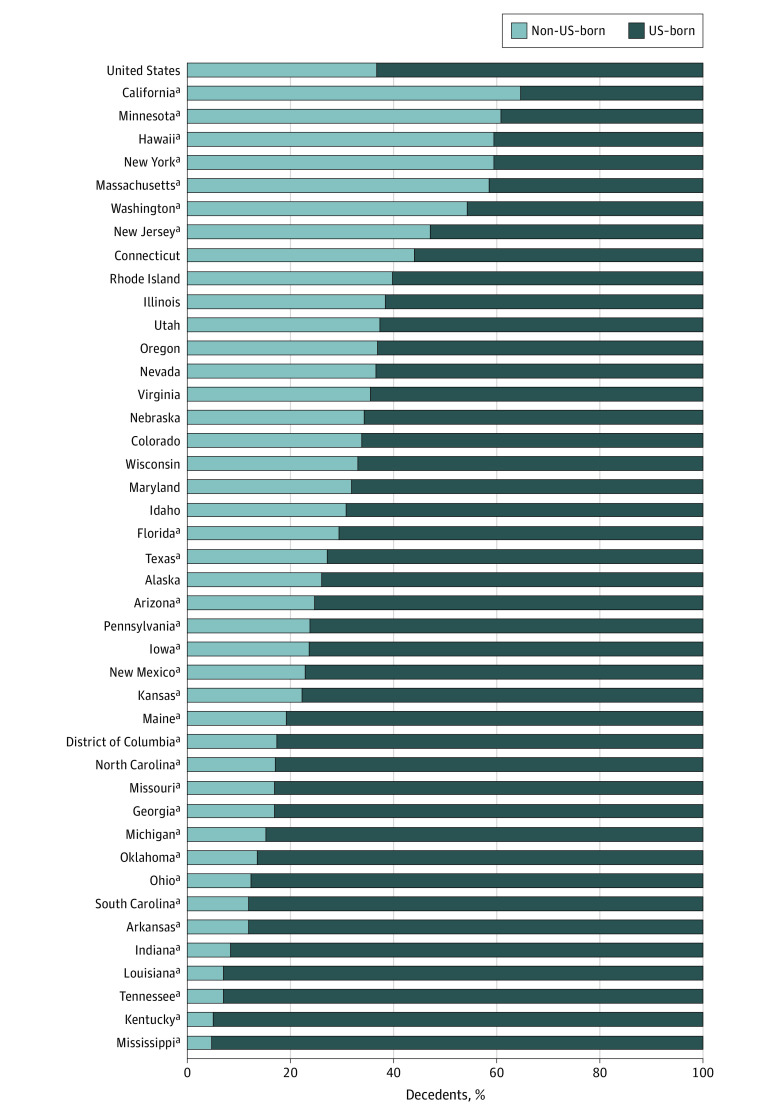
Distribution of US Birthplace Status Among Hepatitis B–Listed Deaths, United States, 2010-2019 Data Source: 2010-2019 US Multiple Cause of Death data, National Vital Statistics System. US birthplace data for Alabama, Delaware, Montana, New Hampshire, North Dakota, South Dakota, Vermont, West Virginia, and Wyoming were not displayed because at least 1 cell (either US-born or non-US–born) had fewer than 10 deaths. Missing values were not included in calculations. ^a^Statistically different based on the 95% CI of the proportion of hepatitis B–listed deaths for each state compared with the national distribution.

Among hepatitis B–listed deaths, US-born decedents, compared with decedents not born in the US, were more frequently aged 45 to 64 years (59.8% [95% CI, 58.9%-60.8%] vs 45.9% [95% CI, 44.6%-47.1%]), born during 1945 to 1965 (66.3% [95% CI, 65.4%-67.2%] vs 54.4% [95% CI, 53.2%-55.7%]), non-Hispanic Black (24.7% [95% CI, 23.9%-25.5%] vs 9.1% [95% CI, 8.4%-9.8%]), non-Hispanic White (67.1% [95% CI, 66.2%-68.0%] vs 10.8% [95% CI, 10.1%-11.6%]), and listed with HCV, HIV, or HDV coinfection (39.4% [95% CI, 38.5%-40.3%] vs 8.8% [95% CI, 8.1%-9.5%]) (Table 2). In 21 states and DC, there were significantly higher proportions of US-born decedents with hepatitis B COD than the national distribution (63.3%), ranging from Florida (70.6% [95% CI, 67.9%-73.4%]) to Mississippi (95.2% [95% CI, 92.3%-98.1%]) (Figure 2). Furthermore, 7 states had significantly higher proportions of non-US–born decedents with hepatitis B COD than the national distribution (36.7%), ranging from New Jersey (47.1% [95% CI, 42.4%-51.8%]) to California (64.7% [95% CI, 63.1%-66.3%]). Washington, New Jersey, and Connecticut had an approximately equal distribution of US- and non-US–born decedents.

**Table 2.** Distribution of Sociodemographic Characteristics, Age at Death, and Underlying Cause of Death by Birthplace of Decedents With Hepatitis B–Listed Deaths, United States, 2010-2019^a^

Category	US-born			Non-US–born				*P* value^c^
	No.	Proportion (95% CI)	Age at death, median (IQR), y	No.	Proportion (95% CI)	Age at death, median (IQR), y	*P* value^b^	
Total	10 823	100	59.0 (53.0-67.0)	6285	100	62.0 (53.0-72.0)	NA	<.001
Sex								
Men	7951	73.5 (72.6-74.3)	59.0 (53.0-66.0)	4534	72.1 (71.0-73.2)	61.0 (52.0-70.0)	.06	<.001
Women	2872	26.5 (25.7-27.4)	60.0 (52.0-69.0)	1751	27.9 (26.8-29.0)	67.0 (57.0-77.0)		<.001
Age, y								
0-34	210	1.9 (1.7-2.2)	32.0 (29.0-33.0)	156	2.5 (2.1-2.9)	31.0 (29.0-33.0)	<.001	.12
35-44	734	6.8 (6.3-7.3)	41.0 (39.0-43.0)	488	7.8 (7.1-8.5)	41.0 (38.0-43.0)		.11
45-64	6476	59.8 (58.9-60.8)	57.0 (52.0-60.0)	2882	45.9 (44.6-47.1)	57.0 (52.0-61.0)		.57
≥65	3403	31.4 (30.6-32.3)	72.0 (67.0-79.0)	2759	43.9 (42.7-45.1)	74.0 (69.0-80.0)		<.001
Year of birth								
After 1965	1679	15.5 (14.8-16.2)	44.0 (39.0-47.0)	1037	16.5 (15.6-17.4)	43.0 (38.0-47.0)	<.001	<.001
1945-1965	7174	66.3 (65.4-67.2)	59.0 (55.0-63.0)	3421	54.4 (53.2-55.7)	60.0 (56.0-65.0)		<.001
Before 1945	1970	18.2 (17.5-18.9)	77.0 (73.0-83.0)	1827	29.1 (27.9-30.2)	78.0 (74.0-83.0)		.05
Race and ethnicity								
American Indian or Alaska Native, non-Hispanic	125	1.2 (1.0-1.4)	58.0 (49.0-65.0)	4	0.1 (0.0-0.2)^f^	62.0 (51.0-68.0)	<.001	.62
Asian or Pacific Islander, non-Hispanic	170	1.6 (1.4-1.8)	59.0 (43.0-69.0)	4263	68.0 (66.8-69.2)	63.0 (54.0-73.0)		<.001
Black, non-Hispanic	2664	24.7 (23.9-25.5)	59.0 (52.0-65.0)	568	9.1 (8.4-9.8)	54.0 (44.0-64.0)		<.001
Hispanic	588	5.5 (5.0-5.9)	58.0 (51.0-65.0)	753	12.0 (11.2-12.8)	61.0 (53.0-70.0)		<.001
White, non-Hispanic	7231	67.1 (66.2-68.0)	60.0 (53.0-68.0)	680	10.8 (10.1-11.6)	65.0 (57.0-75.0)		<.001
HCV, HIV, or HDV coinfection								
Yes	4262	39.4 (38.5-40.3)	57.0 (52.0-62.0)	554	8.8 (8.1-9.5)	62.0 (53.0-69.0)	<.001	<.001
No	6561	60.6 (59.7-61.5)	62.0 (54.0-70.0)	5731	91.2 (90.5-91.9)	63.0 (53.0-72.0)		.02
Hepatitis B COD status								
Listed as the underlying COD	3272	30.2 (29.3-31.1)	59.0 (51.0-68.0)	1846	29.4 (28.2-30.5)	63.0 (54.0-73.0)	.24	<.001
Listed as a contributing COD	7551	69.8 (68.9-70.6)	60.0 (53.0-67.0)	4439	70.6 (69.5-71.6)	62.0 (53.0-72.0)	.24	<.001
Underlying COD when hepatitis B was listed as a contributing COD^d^^,^^e^								
HCV	264	3.5 (3.1-3.9)	58.0 (52.0-62.0)	33	0.7 (0.5-1.0)	62.0 (56.0-73.0)	<.001	.001
Hepatitis A or other viral hepatitis	63	0.8 (0.6-1.1)	58.0 (47.0-64.0)	6	0.1 (0.0-0.3)^f^	60.0 (55.0-63.0)	<.001	.70
Liver-related, alcohol	772	10.2 (9.5-10.9)	56.0 (51.0-61.0)	147	3.3 (2.8-3.9)	56.0 (48.0-63.0)	<.001	.92
Liver-related, nonalcohol	419	5.5 (5.0-6.1)	59.0 (53.0-67.0)	169	3.8 (3.3-4.4)	65.0 (55.0-74.0)	<.001	<.001
Liver cancer	1556	20.6 (19.7-21.5)	61.0 (55.0-67.0)	2384	53.7 (52.2-55.2)	61.0 (52.0-71.0)	<.001	.40
Cancer, except liver cancer	1231	16.3 (15.5-17.2)	62.0 (56.0-69.0)	733	16.5 (15.4-17.6)	64.0 (55.0-72.0)	.76	.23
HIV	616	8.2 (7.6-8.8)	53.0 (46.0-58.0)	82	1.8 (1.5-2.3)	52.0 (42.0-62.0)	<.001	.73
Circulatory	949	12.6 (11.8-13.3)	62.0 (55.0-72.0)	316	7.1 (6.4-7.9)	68.0 (57.5-77.5)	<.001	<.001
Respiratory	345	4.6 (4.1-5.1)	63.0 (57.0-69.0)	69	1.6 (1.2-2.0)	73.0 (62.0-77.0)	<.001	<.001
Diabetes	194	2.6 (2.2-3.0)	61.0 (53.0-68.0)	100	2.3 (1.8-2.7)	65.0 (57.0-76.0)	.28	.001
Genitourinary	97	1.3 (1.0-1.6)	62.0 (53.0-69.0)	58	1.3 (1.0-1.7)	67.0 (54.0-71.0)	.92	.35
Injuries or trauma								
Any	278	3.7 (3.3-4.1)	56.0 (49.0-62.0)	43	1.0 (0.7-1.3)	64.0 (50.0-75.0)	<.001	.009
Drug overdose, alcohol poisoning, suicide, or homicide	174	2.3 (2.0-2.7)	54.0 (46.0-59.0)	14	0.3 (0.2-0.5)^f^	49.5 (41.0-57.0)	<.001	.30
Except drug overdose, alcohol poisoning, suicide, and homicide	104	1.4 (1.1-1.7)	59.0 (52.0-68.5)	29	0.7 (0.4-0.9)	67.0 (56.0-76.0)	<.001	.01
Mental or behavioral disorders	156	2.1 (1.8-2.4)	56.0 (48.5-66.0)	35	0.8 (0.5-1.1)	65.0 (51.0-82.0)	<.001	.05
Digestive, extra-hepatic	129	1.7 (1.4-2.0)	61.0 (53.0-69.0)	64	1.4 (1.1-1.8)	64.0 (56.0-74.5)	.26	.11
Other	482	6.4 (5.8-7.0)	60.5 (53.0-68.0)	200	4.5 (3.9-5.2)	62.0 (53.5-74.0)	<.001	.12

Abbreviations: COD, cause of death; HCV, hepatitis C virus; HDV, hepatitis D virus; NA, not applicable.

^a^ Data Source: 2010-2019 US Multiple Cause of Death data, National Vital Statistics System.

^b^ Assessed differences in the distribution of characteristics between US-born and non-US–born decedents using χ^2^ test of independence.

^c^ Assessed differences in the median age at hepatitis B–listed death between US-born and non-US–born decedents using Kruskal-Wallis test.

^d^ *International Statistical Classification of Diseases and Related Health Problems, Tenth Revision* (*ICD-10*) codes used to define each COD category are provided in the eTable in the Supplement.

^e^ Denominator for proportion calculations of non–hepatitis B conditions listed as the underlying COD was 7551 for US-born decedents and 4439 for non-US–born decedents.

^f^ Proportions where death counts were less than 20 are considered statistically unstable.

During 2010 to 2019, 4969 hepatitis B–listed deaths (28.6%) were also listed with HCV, HIV, or HDV coinfection (only 6 deaths listed HDV infection). Hepatitis B–listed deaths with HCV, HIV, or HDV coinfection, compared with hepatitis B–listed deaths without coinfection, were more frequently aged 45 to 64 years (70.3% [95% CI, 69.1%-71.6%] vs 48.8% [95% CI, 48.0%-49.7%]), born during 1945 to 1965 (75.3% [95% CI, 74.1%-76.5%] vs 56.8% [95% CI, 55.9%-57.7%]), Hispanic (10.0% [95% CI, 9.2%-10.8%] vs 7.0% [95% CI, 6.5%-7.4%]), non-Hispanic Black (24.0% [95% CI, 22.8%-25.2%] vs 17.1% [95% CI, 16.5%-17.8%]), non-Hispanic White (60.4% [95% CI, 59.0%-61.7%] vs 41.0% [95% CI, 40.2%-41.9%]), and US-born (88.5% [95% CI, 87.6%-89.4%] vs 53.4% [95% CI, 52.5%-54.3%]). The proportion of coinfections among hepatitis B–listed deaths in 11 states significantly exceeded the national distribution (28.4%); 41.0% of these deaths occurred in Ohio, Kentucky, Pennsylvania, Tennessee, and West Virginia (eFigure 2 in the Supplement). The proportion of hepatitis B–listed deaths without coinfection significantly exceeded the national distribution (71.6%) in 5 states, ranging from New York (75.7%) to Hawaii (90.1%). Of 6 deaths with HDV coinfection, 4 were among US-born individuals and 5 were men.

Overall, there was no difference in the frequency with which hepatitis B was listed as the UCOD among US- and non-US–born hepatitis B–listed decedents (30.2% [95% CI, 29.3%-31.1%] vs 29.4% [95% CI, 28.2%-30.5%]; *P* = .24) (Table 2). Among US-born decedents with hepatitis B COD, the most frequently listed UCOD was hepatitis B (3272 decedents [30.2%]), followed by liver cancer (1556 decedents [14.4%]) and nonliver cancers (1231 decedents [11.4%]) (Table 3). Among decedents with hepatitis B COD who were not born in the US, the most frequently listed UCOD was liver cancer (2384 decedents [37.9%]), followed by hepatitis B (1846 decedents [29.4%]) and nonliver cancers (733 decedents [11.7%]). For decedents with hepatitis B listed as a CCOD, liver cancer was more frequently listed as the UCOD among non-US–born decedents compared with US-born decedents (53.7% [95% CI, 52.2%-55.2%] vs 20.6% [95% CI, 19.7%-21.5%]; *P* < .001) (Table 2). In contrast, UCOD categories listed more frequently among US-born than non-US–born decedents with hepatitis B COD were HCV infection; other viral hepatitis (primarily hepatitis A); liver-related, alcohol; liver-related, nonalcohol; HIV infection; circulatory; respiratory; injuries or trauma; mental or behavioral disorders, and other (Table 2). Compared with non-US–born decedents with hepatitis B COD, US-born decedents had a significantly younger median age at death for the following UCOD conditions: HCV; liver-related, nonalcohol; circulatory; respiratory; diabetes; and injuries or trauma (Table 2 and Table 3). Irrespective of US birthplace status, hepatitis B–listed decedents had a significantly younger median age at death compared with decedents without hepatitis B COD (Table 3).

**Table 3.** Median Age at Death Among Decedents With and Without Hepatitis B–Listed Deaths, United States, 2010-2019^a^

Cause of death^b^	Decedents without hepatitis B–listed death				Decedents with hepatitis B–listed death				*P* value^e^
					US-born		Non-US–born		
	No (%)	Age at death, median (IQR), y	*P* value^c^	*P* value^d^	No (%)	Age at death, median (IQR), y	No (%)	Age at death, median (IQR), y	
Total	26 697 524 (100)	77.0 (63.0-87.0)	<.001	<.001	10 823 (100)	59.0 (53.0-67.0)	6285 (100)	62.0 (53.0-72.0)	<.001
Hepatitis B COD status									
Listed as the underlying COD	NA	NA	NA	NA	3272 (30.2)	59.0 (51.0-68.0)	1846 (29.4)	63.0 (54.0-73.0)	<.001
Listed as a contributing COD	NA	NA	NA	NA	7551 (69.8)	60.0 (53.0-67.0)	4439 (70.6)	62.0 (53.0-72.0)	<.001
Underlying COD									
Hepatitis C	60 629 (0.2)	59.0 (54.0-65.0)	<.001	.04	264 (2.4)	58.0 (52.0-62.0)	33 (0.5)	62.0 (56.0-73.0)	.001
Hepatitis A or other viral hepatitis	2331 (0.0)	62.0 (53.0-74.0)	<.001	.46	63 (0.6)	58.0 (47.0-64.0)	6 (0.1)^f^	60.0 (55.0-63.0)	.70
Liver-related, alcohol	199 115 (0.7)	57.0 (49.0-63.0)	.17	.52	772 (7.1)	56.0 (51.0-61.0)	147 (2.3)	56.0 (48.0-63.0)	.92
Liver-related, nonalcohol	307 551 (1.2)	65.0 (56.0-74.0)	<.001	.55	419 (3.9)	59.0 (53.0-67.0)	169 (2.7)	65.0 (55.0-74.0)	<.001
Liver cancer	246 854 (0.9)	68.0 (60.0-77.0)	<.001	<.001	1556 (14.4)	61.0 (55.0-67.0)	2384 (37.9)	61.0 (52.0-71.0)	.40
Cancer, except liver cancer	5 789 763 (21.7)	72.0 (63.0-82.0)	<.001	<.001	1231 (11.4)	62.0 (56.0-69.0)	733 (11.7)	64.0 (55.0-72.0)	.23
HIV	65 010 (0.2)	51.0 (43.0-59.0)	.009	.56	616 (5.7)	53.0 (46.0-58.0)	82 (1.3)	52.0 (42.0-62.0)	.73
Circulatory	8 242 400 (30.9)	81.0 (68.0-89.0)	<.001	<.001	949 (8.8)	62.0 (55.0-72.0)	316 (5.0)	68.0 (57.5-77.5)	<.001
Respiratory	2 621 069 (9.8)	79.0 (70.0-87.0)	<.001	<.001	345 (3.2)	63.0 (57.0-69.0)	69 (1.1)	73.0 (62.0-77.0)	<.001
Diabetes	784 356 (2.9)	73.0 (63.0-83.0)	<.001	<.001	194 (1.8)	61.0 (53.0-68.0)	100 (1.6)	65.0 (57.0-76.0)	.001
Genitourinary	675 927 (2.5)	81.0 (70.0-88.0)	<.001	<.001	97 (0.9)	62.0 (53.0-69.0)	58 (0.9)	67.0 (54.0-71.0)	.35
Injuries or trauma									
Any	2 104 208 (7.9)	49.0 (32.0-68.0)	<.001	<.001	278 (2.6)	56.0 (49.0-62.0)	43 (0.7)	64.0 (50.0-75.0)	.009
Drug overdose, alcohol poisoning, suicide, or homicide	1 113 812	42.0 (30.0-55.0)	<.001	.10	174	54.0 (46.0-59.0)	14	49.5 (41.0-57.0)	.30
Except drug overdose, alcohol poisoning, suicide, and homicide	990 396	63.0 (38.0-83.0)	.48	.27	104	59.0 (52.0-68.5)	29	67.0 (56.0-76.0)	.01
Mental or behavioral disorders	1 386 367 (5.2)	87.0 (81.0-92.0)	<.001	<.001	156 (1.4)	56.0 (48.5-66.0)	35 (0.6)	65.0 (51.0-82.0)	.05
Digestive, extra-hepatic	512 486 (1.9)	80.0 (67.0-88.0)	<.001	<.001	129 (1.2)	61.0 (53.0-69.0)	64 (1.0)	64.0 (56.0-74.5)	.11
Other	3 699 458 (13.9)	81.0 (66.0-89.0)	<.001	<.001	482 (4.5)	60.5 (53.0-68.0)	200 (3.2)	62.0 (53.5-74.0)	.12

Abbreviations: COD, cause of death; NA, not applicable.

^a^ Data Source: 2010-2019 US multiple cause-of-death data, National Vital Statistics System.

^b^ *International Statistical Classification of Diseases and Related Health Problems, Tenth Revision* (*ICD-10*) codes used to define each COD category are provided in the eTable in the Supplement.

^c^ Differences in median age at death between decedents who did not have hepatitis B listed as a COD and US-born decedents who had hepatitis B listed as a contributing COD assessed using Kruskal-Wallis test.

^d^ Differences in median age at death between decedents who did not have hepatitis B listed as a COD and non-US–born decedents who had hepatitis B listed as a contributing COD assessed using Kruskal-Wallis test.

^e^ Differences in median age at death between US-born and non-US–born decedents who had hepatitis B listed as a COD assessed using Kruskal-Wallis test.

^f^ Proportions where death counts were fewer than 20 are considered statistically unstable.

### Hepatitis B–Listed Death Rate Changes From 2000 to 2009 vs 2010 to 2019

Compared with 2000 to 2009, during 2010 to 2019, the national hepatitis B–listed death rate declined by 19.0% (*P* < .001) (Table 1). Although significant declines in death rates occurred in 14 states (largest significant declines in Connecticut [45.8%; *P* = .005] and Pennsylvania [40.9%; *P* = .005]), significant increases were observed in West Virginia (83.8%; *P* = .005), and Kentucky (69.4%; *P* = .005) (Table 1).

## Discussion

In this cross-sectional study assessing hepatitis B–listed deaths in the US, we observed a hepatitis B–listed death rate of 0.47 deaths per 100 000 population during 2010 to 2019, which represented significant declines for the US as a whole and for 14 states compared with 2000 to 2009. However, significant increases in hepatitis B–listed death rates were observed in West Virginia and Kentucky. These states also experienced the highest death rates from all causes in 2017.^28^ In all, areas with significantly higher hepatitis B–listed death rates than the national hepatitis B–listed death rate were primarily in the coastal and Appalachian regions. Compared with the national median age at hepatitis B–listed death, significantly younger median age at death occurred among decedents of 5 Appalachian states, where most decedents were US-born and approximately one-third had HCV or HIV coinfection. In contrast, a significantly older median age at death was observed among California decedents, who were predominantly not born in the US. Hepatitis B–listed deaths for persons not born in the US occurred primarily in West and East Coast states with large immigrant populations from hyperendemic areas.^29^ Non-US–born decedents more frequently had an older median age at death compared with the national median age at hepatitis B–listed death.

Nearly two-thirds of decedents with hepatitis B–listed deaths were born in the US; in 21 states and DC, the fraction of US-born decedents exceeded the national distribution. US-born decedents were more frequently coinfected with HCV or HIV than those not born in the US. During 2010 to 2019, only 6 decedents had HDV coinfection listed. Given that HDV testing is conducted inconsistently in the United States,^30^ it is likely that HDV coinfection deaths have been continually underreported.

We found no difference in the frequency with which hepatitis B was listed as the UCOD among US- and non-US–born decedents. However, when hepatitis B was listed instead as a CCOD, decedents not born in the US more often had liver cancer listed as the UCOD, whereas US-born decedents more often had UCOD conditions that included HCV infection, other viral hepatitis, HIV infection, alcoholic liver disease, respiratory and circulatory conditions, and drug overdose, alcohol poisoning, suicide, or homicide. A recent vital statistics analysis^31^ found that since 2014, these conditions also contributed to decreases in the overall US life expectancy, especially in areas where we observed high proportions of US-born hepatitis B–listed deaths and younger median ages at death, such as the Ohio River Valley and Appalachian region.

As chronic hepatitis B is a heterogeneous condition with a highly variable and unpredictable course, all persons with chronic infection require continual lifelong assessment, as a sizable proportion at some point may be indicated for hepatitis B antiviral therapy.^32^ Whether infected at birth or later in life, all persons with chronic hepatitis B need continual, lifelong medical care to monitor disease activity to determine whether and when antiviral treatment is indicated, and to undergo periodic surveillance for hepatocellular carcinoma as recommended.^32^ Irrespective of birthplace status or whether hepatitis B was listed as the UCOD or a CCOD, we found that decedents with hepatitis B COD had a significantly younger median age at death than decedents without hepatitis B listed as a COD. Most decedents with hepatitis B listed as a COD, regardless of birth status, had liver-related UCOD.

### Limitations

This study has some limitations. Since this analysis hinged exclusively on the interpretation of MCOD data, its principal limitations were the use of data based on clinician judgment about causes and relative importance of conditions leading to death. Death certificates have been shown to have high rates of error, particularly as they pertain to chronic liver disease.^23,24^ Studies using medical records to validate death certificate COD have consistently shown that mortality data greatly underestimates the true hepatitis B mortality burden, even when persons with hepatitis B infection had a liver-related COD.^1,24,33^ Although findings from this study may be underestimated, they nonetheless support the consideration of CCOD in addition to the UCOD, and, more importantly, attest to its capacity to permit identification of the various conditions—especially those leading to early death—afflicting persons with chronic hepatitis B in the United States.^2,23,24^ Next, we were not able to calculate national age-adjusted hepatitis B–listed death rates in the US-born and non-US–born populations because those population-specific denominators are not available in the US Census population data. Additionally, American Indian and Alaska Native persons are often misclassified as other racial and ethnic groups on death certificates,^26^ resulting in underreporting of conditions among American Indian and Alaska Native persons. Furthermore, geographic characterizations among non-US–born persons should be interpreted with caution, as they may change their residence often before settling.

## Conclusions

The findings of this cross-sectional study suggest that in addition to diagnosis and management of hepatitis B among non-US–born persons, US-born persons with chronic infection, who constituted approximately two-thirds of all hepatitis B–listed deaths, may also require diagnosis and management of viral coinfections, respiratory and cardiovascular conditions, nonviral liver disease, and addiction-related sequelae. Further reduction of hepatitis B mortality will require access to hepatitis B-related care that is continual and life-long. Universal hepatitis B screening in adults is expected to identify more persons with hepatitis B in need of linkage to comprehensive and routine medical care.

## Notes

Supplement. **eFigure 1.** Distribution of Deaths Listed With Hepatitis B, Hepatitis C, HIV, and Hepatitis D, United States, 2010-2019 **eFigure 2.** Distribution of Hepatitis C Virus (HCV), HIV, or Hepatitis D Virus (HDV) Coinfection Listed as a Cause of Death Among Hepatitis B–Listed Deaths, United States, 2010-2019 **eTable.** Cause of Death Categories and Corresponding *International Statistical Classification of Diseases and Related Health Problems, Tenth Revision* (*ICD-10*) Codes
