# Supplementary material for: Regional Differences in Mortality Rates and Characteristics of Decedents With Hepatitis B Listed as a Cause of Death, United States, 2000-2019
Source: JAMA Netw Open. 2022 Jun 28;5(6):e2219170. doi: 10.1001/jamanetworkopen.2022.19170 (PMC9240905; doi:10.1001/jamanetworkopen.2022.19170)
Supplement: Supplement. — eFigure 1. Distribution of Deaths Listed With Hepatitis B, Hepatitis C, HIV, and Hepatitis D, United States, 2010-2019 eFigure 2. Distribution of Hepatitis C Virus (HCV), HIV, or Hepatitis D Virus (HDV) Coinfection Listed as a Cause of Death Among Hepatitis B–Listed Deaths, United States, 2010-2019 eTable. Cause of Death Categories and Corresponding International Statistical Classification of Diseases and Related Health Problems, Tenth Revision (ICD-10) Codes [file jamanetwopen-e2219170-s001.pdf]

Supplemental Online Content

Ly KN, Yin S, Spradling PR. Regional differences in mortality rates and characteristics of decedents with hepatitis B listed as a cause of death, United States, 2000-2019. *JAMA Netw Open*. 2022;5(6):e2219170. doi:10.1001/jamanetworkopen.2022.19170

eFigure 1. Distribution of Deaths Listed With Hepatitis B, Hepatitis C, HIV, and Hepatitis D, United States, 2010-2019

eFigure 2. Distribution of Hepatitis C Virus (HCV), HIV, or Hepatitis D Virus (HDV) Coinfection Listed as a Cause of Death Among Hepatitis B–Listed Deaths, United States, 2010-2019

eTable. Cause of Death Categories and Corresponding *International Statistical Classification of Diseases and Related Health Problems, Tenth Revision* (*ICD-10*) Codes

This supplemental material has been provided by the authors to give readers additional information about their work.

© 2022 Ly KN et al. *JAMA Network Open.*

eFigure 1. Distribution of Deaths Listed With Hepatitis B, Hepatitis C, HIV, and Hepatitis D, United States, 2010-2019

Data Source: 2010-2019 US Multiple Cause of Death data, National Vital Statistics System. Venn diagram was generated using Rstudio, version 1.3.1056 (Boston, MA).

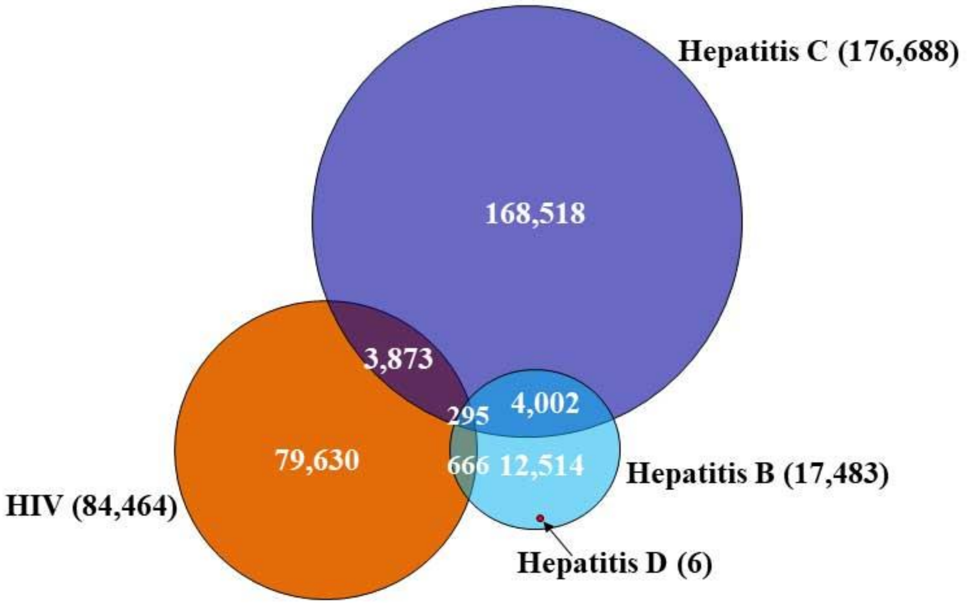

© 2022 Ly KN et al. *JAMA Network Open.*

eFigure 2. Distribution of Hepatitis C Virus (HCV), HIV, or Hepatitis D Virus (HDV) Coinfection Listed as a Cause of Death Among Hepatitis B–Listed Deaths[a], United States, 2010-2019

Data Source: 2010-2019 US Multiple Cause of Death data, National Vital Statistics System.

Data for Montana, North Dakota, South Dakota, and Wyoming were not displayed because at least 1 cell (either not listed with HCV, HIV, or HDV coinfection or listed HCV, HIV, or HDV infection) had fewer than 10 deaths.

[a] Statistically different based on the 95% confidence interval of the proportion of hepatitis B-listed deaths for each state compared to the national distribution. Hepatitis B-listed deaths were defined as hepatitis B ICD-10 codes B16, B17.0, B18.0, and B18.1 listed as the underlying cause or one of the contributing causes of death. Coinfection status was determined by having HCV (ICD-10 codes B17.1 or B18.2), HIV (ICD-10 codes B20–B24), or HDV (ICD-10 codes B16.0, B16.1, B17.0, and B18.0) coinfection listed as a cause of death. Six deaths listed HDV infection.

© 2022 Ly KN et al. *JAMA Network Open.*

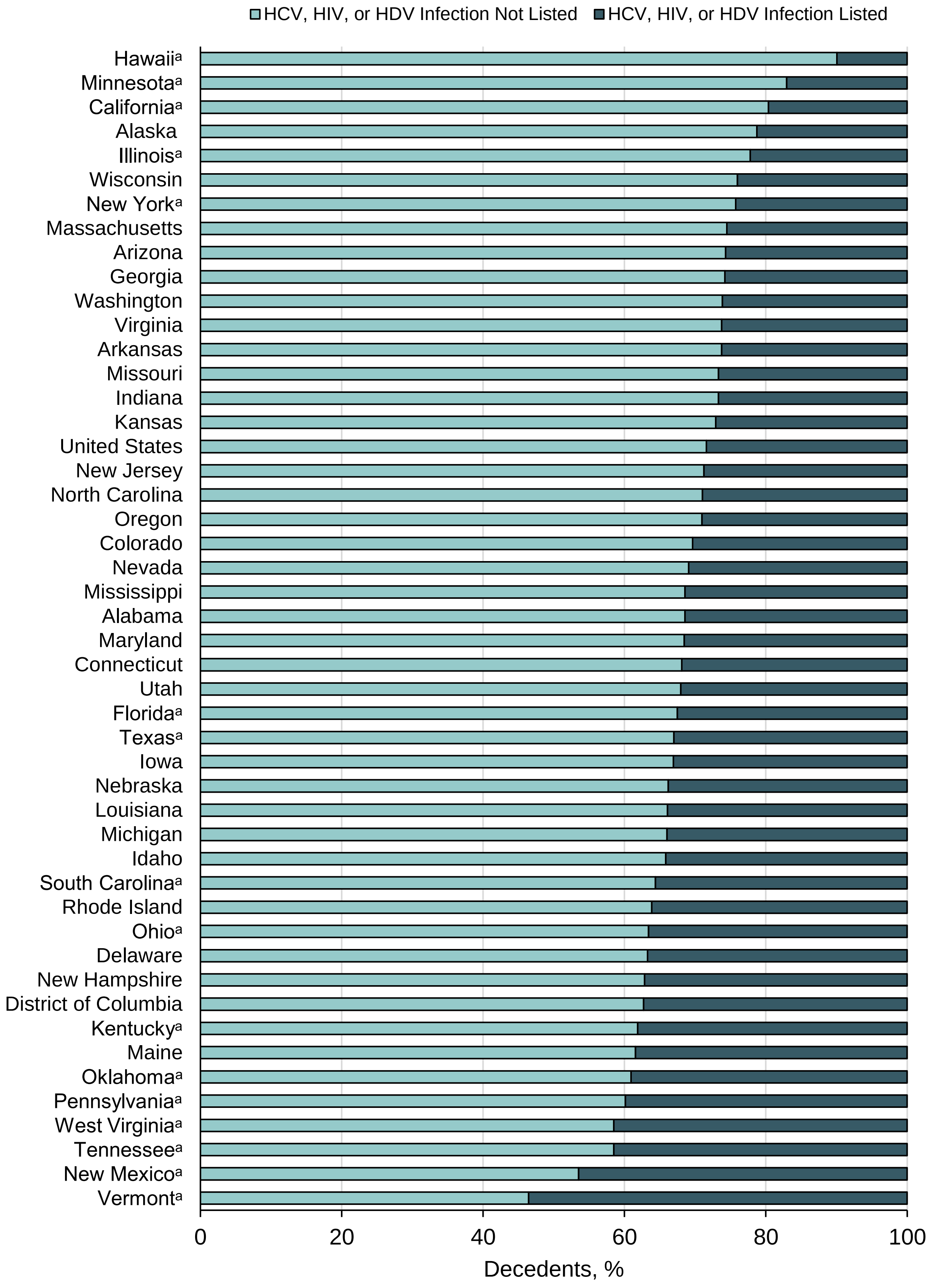

© 2022 Ly KN et al. *JAMA Network Open.*

eTable. Cause of Death Categories and Corresponding *International Statistical Classification of Diseases and Related Health Problems, Tenth Revision* (*ICD-10*) Codes

Cause of Death Category	ICD-10 Codes
Hepatitis B	B16, B17.0, B18.0, or B18.1
Hepatitis C	B17.1 or B18.2
Hepatitis A or other viral hepatitis	B15, B17.2, B17.8, B17.9, B18.8, B18.9, B19, or B94.2
Liver-related, alcohol	K70
Liver-related, nonalcohol	K71-K77
Liver cancer	C22 or D37.6
Cancer, except liver cancer	C00-C97 (except C22) or D37-D48 (except D37.6)
HIV infection	B20-B24
Circulatory	I00-I99
Respiratory	J00-J99
Diabetes	E10-E14
Genitourinary	N00-N99
Injuries or trauma	
Any	S00-S99, T00-T98, V01-V99, W00-W99, X00-X99, or Y00-Y36
Drug overdose, alcohol poisoning, suicide, or homicide	X40-X45 or X60-Y15
Except drug overdose, alcohol poisoning, suicide, and homicide	S00-S99, T00-T98, V01-V99, W00-W99, X00-X39, X46-X59, or Y16-Y36
Mental or behavioral disorders	F00-F99
Digestive, extra-hepatic	K00-K67 or K80-K93
Other	A00-A99, B00-B09, B25-B99 (except B94.2), D00-D36, D50-D89, E00-E07, E15-E99, G00-G99, H00-H95, L00-L99, M00-M99, O00-O99, P00-P96, Q00-Q99, R00-R99, U00-U85, Y40-Y98, or Z00-Z99

Abbreviation: ICD-10, International Classification of Diseases, Tenth Revision.

Data Source: World Health Organization. ICD-10 Version: 2019. Available at: https://icd.who.int/browse10/2019/en#/.

© 2022 Ly KN et al. *JAMA Network Open.*
